# High variance in reproductive success generates a false signature of a genetic bottleneck in populations of constant size: a simulation study

**DOI:** 10.1186/1471-2105-14-309

**Published:** 2013-10-16

**Authors:** Sean M Hoban, Massimo Mezzavilla, Oscar E Gaggiotti, Andrea Benazzo, Cock van Oosterhout, Giorgio Bertorelle

**Affiliations:** 1 Department of Life Sciences and Biotechnology, University of Ferrara, via Borsari 46, Ferrara I-44121, Italy; 2 National Institute for Mathematical and Biological Synthesis (NIMBios), The University of Tennessee, Knoxville, TN 37996, USA; 3 Institute for Maternal and Child Health, IRCCS, University of Trieste, via dell’Istrai 65, Trieste I-34137, Italy; 4 School of Biology, Scottish Oceans Institute, University of St Andrews, St Andrews, Fife KY16 8LB, UK; 5 School of Environmental Sciences, University of East Anglia, Norwich Research Park, Norwich NR4 7TJ, UK

**Keywords:** Conservation, Heterozygosity excess, M-ratio, MSVAR, FPR, Sweepstakes reproduction, Type I error, Variance in reproductive success

## Abstract

**Background:**

Demographic bottlenecks can severely reduce the genetic variation of a population or a species. Establishing whether low genetic variation is caused by a bottleneck or a constantly low effective number of individuals is important to understand a species’ ecology and evolution, and it has implications for conservation management. Recent studies have evaluated the power of several statistical methods developed to identify bottlenecks. However, the false positive rate, i.e. the rate with which a bottleneck signal is misidentified in demographically stable populations, has received little attention. We analyse this type of error (type I) in forward computer simulations of stable populations having greater than Poisson variance in reproductive success (i.e., variance in family sizes). The assumption of Poisson variance underlies bottleneck tests, yet it is commonly violated in species with high fecundity.

**Results:**

With large variance in reproductive success (*V*_*k*_ ≥ 40, corresponding to a ratio between effective and census size smaller than 0.1), tests based on allele frequencies, allelic sizes, and DNA sequence polymorphisms (heterozygosity excess, M-ratio, and Tajima’s *D* test) tend to show erroneous signals of a bottleneck. Similarly, strong evidence of population decline is erroneously detected when ancestral and current population sizes are estimated with the model based method MSVAR.

**Conclusions:**

Our results suggest caution when interpreting the results of bottleneck tests in species showing high variance in reproductive success. Particularly in species with high fecundity, computer simulations are recommended to confirm the occurrence of a population bottleneck.

## Background

Demographic fluctuations, including changes in population size and growth rate, are common events in natural populations. Severe population size declines (bottlenecks), however, may have detrimental consequences including increased inbreeding, decreased adaptive potential, increased disease susceptibility, lowered fecundity, and disruption in expression of quantitative traits [1-3]. As bottlenecks often affect long-term fitness and population viability, or change the balance of drift and selection, they are key events in a species' evolutionary history, and a principal concern for endangered species [4].

Bottlenecks may leave a population genetic signature, such as decreases in number of alleles and heterozygosity, and loss of rare alleles [5,6]. These signatures can be easily detected when temporal samples are available (e.g. museum specimens or fossil remains), so that contemporary genetic variation can be compared to historic levels. A bottleneck, however, may also leave specific signatures in current genetic variation, distinct from those in populations having a history of small and constant size. Indeed, several methods for detecting genetic bottlenecks in the absence of information about historical sizes and in absence of pre-bottleneck genetic samples exist [7-10]. Genetic methods for bottleneck detection are useful because: (1) historical (and current) census sizes are rarely known; (2) even when census size (*N*_*c*_) is known, cryptic bottlenecks (change in effective size, *N*_*e*_, without change in *N*_*c*_) may occur; and (3) bottleneck outcomes are highly stochastic, meaning that genetic diversity following the bottleneck is somewhat unpredictable even when the demographic history is known [11,12]. It is therefore important to evaluate the statistical performance of these methods, especially as these tests are key components of many evolutionary, molecular ecology, and conservation genetic studies [13-16].

Previous investigations have demonstrated that the statistical power of these tests is highest when the bottleneck is severe or prolonged, and when many loci are used. In addition, factors such as the mutation model and the rate of post-bottleneck recovery may also play an important role [9,13,16-18]. Also, the methods do not always show similar power. For example, the heterozygosity excess test [9] has low power after rapid recovery [17] whereas the M-ratio test [10] remains effective, and the heterozygosity-excess test is weak unless the population is reduced to some tens of individuals [19]. Bottleneck signals are also weakened if the bottleneck occurred gradually, or if the population recovered to its pre-bottleneck population size [16]. Numerous empirical studies have failed to detect a genetic signal even when a moderate or strong demographic bottleneck is known to have occurred [4,11,20], showing empirically that the power of such tests can be limited.

A lack of statistical power in bottleneck tests may result in an underestimation of the extinction risk. On the other hand, identifying bottleneck signatures when they have not occurred may represent a complementary risk [18,21], yet this remains an often overlooked aspect of studies employing these methods. Controlling type I error rate (FPR, False Positive Rate) is important, particularly given that resources towards conservation tend to be limited [22]. Type I error could result in incorrect population protection status or unwarranted, ineffective, or even detrimental in-situ management interventions (e.g., translocations, augmentations). Therefore, an understanding of type I error in realistic situations is essential to properly use and interpret results from these methods.

Investigations of type I error of bottleneck detection methods are few, and have mostly concerned mutation models in microsatellite markers. For example, the probability of type I error can be substantial or extreme (from 40 to 100%) when the wrong mutation model is assumed or when multi-step mutations occur [21]. Also, assuming the wrong population-mutation parameter theta (θ = 4*N*_*e*_*μ,* where *μ* is the mutation rate) in the M-ratio test may result in either type I or type II errors, depending on whether the assumed θ is larger or smaller than the actual value [23]. Remarkably, in spite of frequent use of bottleneck tests, and the conservation decisions that are based on them, little is known about their type I error rates when assumptions of the biological model are violated. For example, the influence of mating patterns [13], age structure [14], and reproductive success [21,22] is rarely known.

Here we focus on type I error rates that may arise in bottleneck tests when the variance of reproductive success (hereafter *V*_*k*_) is larger than the Poisson variance assumed by simple models underlying the bottleneck detection methods. Larger than Poisson *V*_*k*_ could cause strong intergenerational genetic drift, because it introduces additional stochasticity (e.g. unaccounted loss of alleles) when only few parents contribute to the next generation [24]. When extreme, this process has been referred to as Sweepstakes Reproductive Success (SRS), in which many individuals “lose” and produce zero or very few offspring, while one or a few individuals "win" and produce many offspring [25,26]. Such extreme reproductive variance can be caused by complete or near-complete dominance of one pair, or positively correlated sibling survival, in which all offspring of a particular brood survive or perish [24,25]. Variance can also be extreme when only one male contributes offspring [12]. Large *V*_*k*_ reduces the *N*_*e*_*/N*_*c*_ ratio, which may explain *N*_*e*_*/N*_*c*_ in the order of 10^-2^ - 10^-3^ observed in many amphibians, fish, marine invertebrates, and plants. Even more extreme *N*_*e*_*/N*_*c*_ ratios, as low as 10^-3^ to 10^-5^, have been reported for lobster, cod, red drum, and oyster [27]. “Chaotic” genetic differences at small geographic scale and at different time intervals often observed in marine organisms has been explained as relating to high *V*_*k*_[26].

Theoretically, the relationship between *V*_*k*_ and the *N*_*e*_*/N*_*c*_ ratio has been derived under different models [25,28,29], and hence, an increase in *V*_*k*_ can be converted into a predictable reduction in *N*_*e*_. However, the effect of *V*_*k*_ on the shape of a coalescent tree and on the relationship between different genetic diversity measures (which are the basis for bottleneck testing) have not been investigated [27]. In particular, it remains to be elucidated whether analysis of genetic data from species with large *V*_*k*_ will show signature of small but constant size, or whether large *V*_*k*_ results in a false signal of a genetic bottleneck. Here we investigate this question for different combinations of *N*_*e*_ and *V*_*k*_ values, using simulated data to estimate type I errors in two tests commonly applied to microsatellite data to detect bottlenecks, the M-ratio [10] and the heterozygosity test [9], and when ancestral and current population size are estimated to infer bottlenecks with the MSVAR method [7]. We also consider the effects of *V*_*k*_ on the Tajima’s *D* test [30], which is used to detect selection as well as deviations from demographic stability in DNA sequence polymorphisms. All these tests assume stable populations with Poisson-distributed family sizes, i.e. *V*_*k*_ = 2.

## Methods

Genetic variation data were generated by simulating demographically stable populations with different effective size (*N*_*e*_) and different variance in reproductive success (*V*_*k*_). For each combination of parameters, 100 replicates were generated. Each data set, consisting of 15 microsatellite markers, was analysed with the M-ratio and the heterozygosity excess tests, and with the MSVAR method. The fraction of replicates significantly supporting a bottleneck can be considered as an estimate of the FPR (false positive rate), i.e., the type I error rate. Then a smaller set of simulations was used to analyse two additional markers (microsatellite loci with constrained allelic range and DNA sequence polymorphisms).

### Generating the primary set of synthetic data

**Figure 1:**
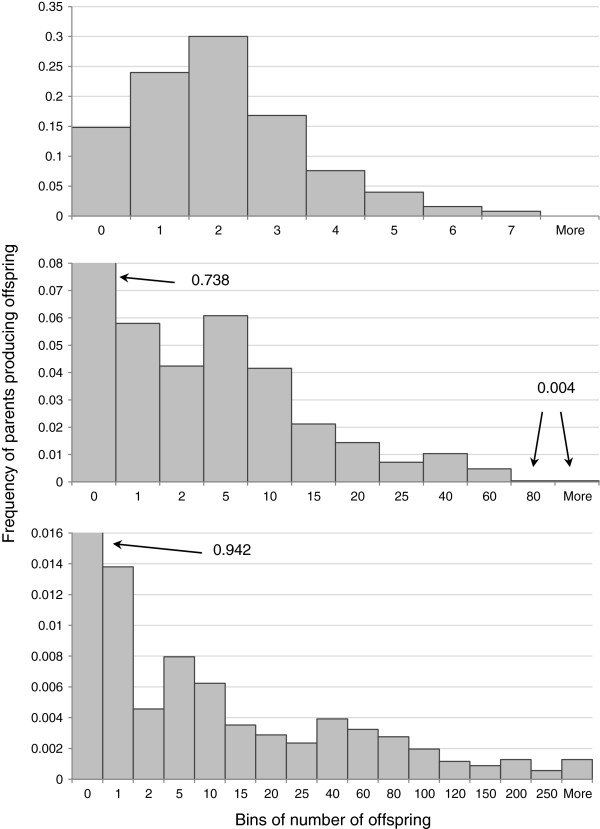
**An example of the distribution of offspring per parent in the simulations.** The three panels correspond to the distributions obtained in simulations with *V*_*k*_ =2 (top), *V*_*k*_ = 40 (middle), and *V*_*k*_ = 400 (bottom).

The software simuPOP [31] was used to generate the virtual data. simuPOP is an individual-based, forward-in-time simulator that uses the flexible scripting language Python to allow operators that control sex ratio, number of offspring produced etc., and is one of few simulators to allow such options [32]. Random mating of individuals and family sizes with different distributions (i.e., *V*_*k*_) can be simulated straightforwardly. We analysed 16 combinations of *N*_*e*_ (50, 500, 2500 and 5000) and *V*_*k*_ (2, 40, 400, and 2000). Population size was assumed to be constant, and the mean number of offspring per mating was always equal to two. In order to obtain the same *N*_*e*_ for different *V*_*k*_ values, the census sizes required in the simulations were computed using the approximate relationship *N*_*e*_*/N*_*c*_ = 4/(*V*_*k*_ +2) [25,33]. When *V*_*k*_ =2, family sizes were Poisson distributed (as assumed by most population genetics models) and the ratio *N*_*e*_*/N*_*c*_ =1. For larger *V*_*k*_, we used a modified gamma distribution of family sizes with decimal values rounded down to the nearest integer (resulting in a discrete distribution approximating a negative binomial, Figure 1). This choice allowed us to maintain the average number of offspring per mating equal to two while producing a long right tail in the distribution. The *V*_*k*_ values of 40, 400, and 2000 correspond approximately to *N*_*e*_*/N*_*c*_ equal to 0.1, and 0.01, 0.002, respectively.

Fifteen neutral, independent microsatellites evolving under a strict stepwise mutation model with mutation rate *μ*=5×10^-4^ were considered. Mutation-drift equilibrium was obtained by running simulations for *N*_*e*_ generations, starting from individuals with a Dirichelet distribution of allele frequencies. After verifying that the population had reached a stable equilibrium confirmed by the convergence of the number of alleles (*K)*, the expected heterozygosity (*H*_*e*_), and the inbreeding coefficient *F*_*is*_, 50 individuals were randomly sampled and analysed using ARLEQUIN v3.5 [34] for the summary statistics noted above, the M-ratio and the Tajima’s *D* tests, and using BOTTLENECK v.1.2.1 [9] for the heterozygote excess test, and MSVAR v. 1.3 for the estimation of current vs. ancestral population sizes [7].

### Additional simulations

Some specific situations were investigated using additional simulations. First we simulated microsatellite markers where the maximum number of alleles is limited to five, to represent expressed (EST) microsatellites which tend to have a limited allelic range; a restricted allele range may affect the M-ratio. Second we simulated DNA sequences of 500 base pairs evolving under an infinite site mutation model with mutation rate *μ*=10^-7^ per site per generation. These simulations were conducted to understand whether the spurious signal of a bottleneck produced by *V*_*k*_ > 2 is specific to microsatellites markers, or whether a similar signal would be found when Single Nucleotide Polymorphisms (SNPs) are considered.

### Bottleneck tests

Microsatellite data was analysed first with the commonly used M-ratio test [10] and heterozygosity excess test [9]. The M-ratio test is based on the frequency distribution of allelic sizes, which is expected to have gaps after a bottleneck due to stochastic loss of rare alleles. The M-ratio is computed in each data set as simply the ratio of the number of occupied allelic states divided by the number of possible allelic states (e.g. the range). Evidence of deviation from the null hypothesis of demographic stability can be concluded in one of two ways: if the observed value is lower than a simple threshold criteria (M-ratio < 0.68 [10], which is widely used as a “rule of thumb” in conservation genetics) or if the observed value is lower than a critical value, determined by reconstructing the null distribution of M using 1000 coalescent simulations. The coalescent simulations used to generate this null distribution assume by definition *V*_*k*_ = 2. Also we set the parameters *N*_*e*_ and *μ* to the values used in producing the corresponding data sets. Throughout the paper, we will call M-ratio_ft_ test the approach based on the fixed threshold, and M-ratio_sim_ test the approach that uses simulations to compute the critical value. The heterozygosity excess test is based on a relationship between heterozygosity and number of alleles, which is predicted to deviate from theoretical expectations after a bottleneck because the former decreases more slowly than the latter. Statistical significance for this test is computed using the Wilcoxon’s signed rank test to compare the expected heterozygosity calculated from the data (H_e_) to an expected heterozygosity based on the number of alleles present (H_a_) [9], where H_a_ is computed by simulation using the program BOTTLENECK [35].

We performed also a more sophisticated analysis which is frequently used to detect changes in population size [7]. This analysis uses a full-likelihood model-based approach called MSVAR to infer current and past population sizes as well as other parameters. The method can be used to infer a bottleneck if the ratio of past to current population size is significantly greater than unity. From each MSVAR analysis, the posterior distribution of the ratio between ancient and current population sizes was estimated, and the data set was considered to support the bottleneck hypothesis if less than 5% of this distribution was smaller than 1. For each data set (1600 in total), we recorded the MCMC (Monte Carlo Markov Chain) output 40,000 times every 10,000 steps. The first 10% of steps were discarded as burn in. Means and variances for priors and hyperpriors are reported in the legend of Table 1. In some cases this approach has been shown to be more powerful than the simpler statistics explained above [11], but it also relies on more assumptions (a particular demographic model).

**Table 1:** Simulation results for a population with constant size and standard microsatellite mutations

**N**_**e**_	**V**_**k**_	**N**_**e**_**/N**_**c**_	**H**_**e **_**(SD)**	**K (SD)**	**F**_**is **_**(SD)**	**M-ratio (SD)**	**%P**	**FPR**			
								**M-ratio**_**ft**_	**M-ratio**_**sim**_	**Het excess**	**MSVAR**
50											
	2	1	0.11 (0.17)	1.53 (0.59)	0.00 (0.09)	1.00 (0.03)	48	0.01	0.02	0.01	0.00
	40	0.1	0.07 (0.14)	1.30 (0.52)	-0.03 (0.12)	1.00 (0.00)	27	0.0	0.04	0.02	0.00
	400	0.01	0.05 (0.14)	1.24 (0.45)	-0.01 (0.11)	1.00 (0.03)	23	0.01	0.04	0.10	0.00
	2000	0.002	0.07 (0.13)	1.25 (0.35)	-0.15 (0.17)	0.97 (0.06)	25	0.00	0.17	0.11	0.00
500											
	2	1	0.44 (0.16)	3.08 (0.72)	-0.02 (0.12)	1.00 (0.03)	100	0.0	0.09	0.04	0.00
	40	0.1	0.42 (0.20)	2.74 (0.81)	-0.07 (0.21)	0.98 (0.07)	96	0.03	0.36	0.32	0.62
	400	0.01	0.43 (0.23)	2.91 (1.10)	-0.17 (0.29)	0.87 (0.18)	89	0.21	1.00	0.53	0.97
	2000	0.002	0.44 (0.21)	3.17 (1.20)	-0.19 (0.31)	0.71 (0.21)	88	0.43	1.00	0.54	1.00
2500											
	2	1	0.71 (0.06)	6.3 (1.3)	0.01 (0.05)	0.95 (0.08)	100	0.0	0.03	0.06	0.06
	40	0.1	0.69 (0.1)	5.7 (1.8)	-0.08 (0.11)	0.89 (0.13)	100	0.07	0.51	0.20	0.66
	400	0.01	0.64 (0.09)	4.5 (1.2)	-0.19 (0.13)	0.82 (0.15)	99	0.35	1.00	0.39	0.99
	2000	0.002	0.61 (0.12)	4.2 (1.4)	-0.20 (0.12)	0.69 (0.18)	99	0.49	1.00	0.42	1.00
5000											
	2	1	0.76 (0.08)	7.70 (1.60)	-0.016 (0.08)	0.94 (0.09)	100	0.0	0.05	0.07	0.14
	40	0.1	0.72 (0.09)	6.06 (1.76)	-0.11 (0.17)	0.81 (0.19)	100	0.23	0.93	0.22	0.97
	400	0.01	0.66 (0.13)	4.80 (1.51)	-0.22 (0.16)	0.68 (0.23)	100	0.50	1.00	0.40	1.00
	2000	0.002	0.67 (0.11)	4.90 (1.66)	-0.24 (0.14)	0.66 (0.20)	99	0.58	1.00	0.43	1.00

Mean values of summary statistics (with standard deviations) across 100 replicates are given. The last four columns report the rate of false positives (FPR = type I error) estimated as the fraction of replicates with an M-ratio smaller than the commonly used threshold of 0.68 (M-ratio_ft_), with a M-ratio smaller and the critical value computed by simulation using the same parameter θ = 4*N*_*e*_*μ* used to generate the data (M-ratio_sim_), where a significant (P< 0.05) heterozygoty excess was detected using the program BOTTLENECK, and where a significant difference between ancestral and current population size is detected by MSVAR, respectively. *N*_*e*_ = effective population size; *N*_*c*_ = census population size; *H*_*e*_ = expected heterozygosity; *F* = inbreeding coefficient, estimated as 1-*H*_*o*_/*H*_*e*_, where *H*_*o*_ is the observed heterozygosity; M = M-ratio; %P = fraction of replicates producing a polymorphic locus; the starting values, in the log_10_ scale, for the mean and variance of the prior distributions in MSVAR, are as follows: ancestral size (3,1), current size (3,1), mutation rate ( -3.3,1), time since the decline (2,0.5); means and variances (and their means and variances) of the hyperprior distributions used in MSVAR are as follows: ancestral size (3,1,0,0.5), current size (3,1,0,0.5), mutation rate (-3.3,0.25,0,0.5), time since the decline (2,0.5,0,0.5).

DNA sequences were analysed with the Tajima’s *D* test [30], which is based on the comparison between the average pairwise difference (*π*) and the number of polymorphic sites (*S*). If equilibrium is not reached after a demographic event, negative *D* values are expected under population expansion and positive *D* values are expected under population decline [30]. Tajima’s *D* is commonly used also to detect deviation from neutrality, i.e. the impact of selection on DNA sequences. Statistical significance is computed by simulations, as implemented in Arlequin [34].

## Results

### Primary set of simulations

As expected, the average level of genetic variation (expected heterozygosity, *H*_*e*_, and number of alleles, *K*) increased with increasing *N*_*e*_. The average *H*_*e*_ observed for *V*_*k*_=2 is similar to theoretical predictions [36] which are 0.09, 0.42 and 0.78 for *N*_*e*_ values 50, 500, and 5000. The number of alleles does not have a simple expectation under the single-step mutation model, but the observed values are compatible with other results [37]. When *V*_*k*_ increases, we observe a trend of decreased genetic variation within each set of simulations with the same *N*_*e*_, and this effect is stronger for *K* than for *H*_*e*_. For *V*_*k*_ > 2, populations also appear to deviate from the Hardy-Weinberg equilibrium, with larger observed than expected heterozygosity and consequent negative values of the estimated inbreeding coefficient.

**Figure 2:**
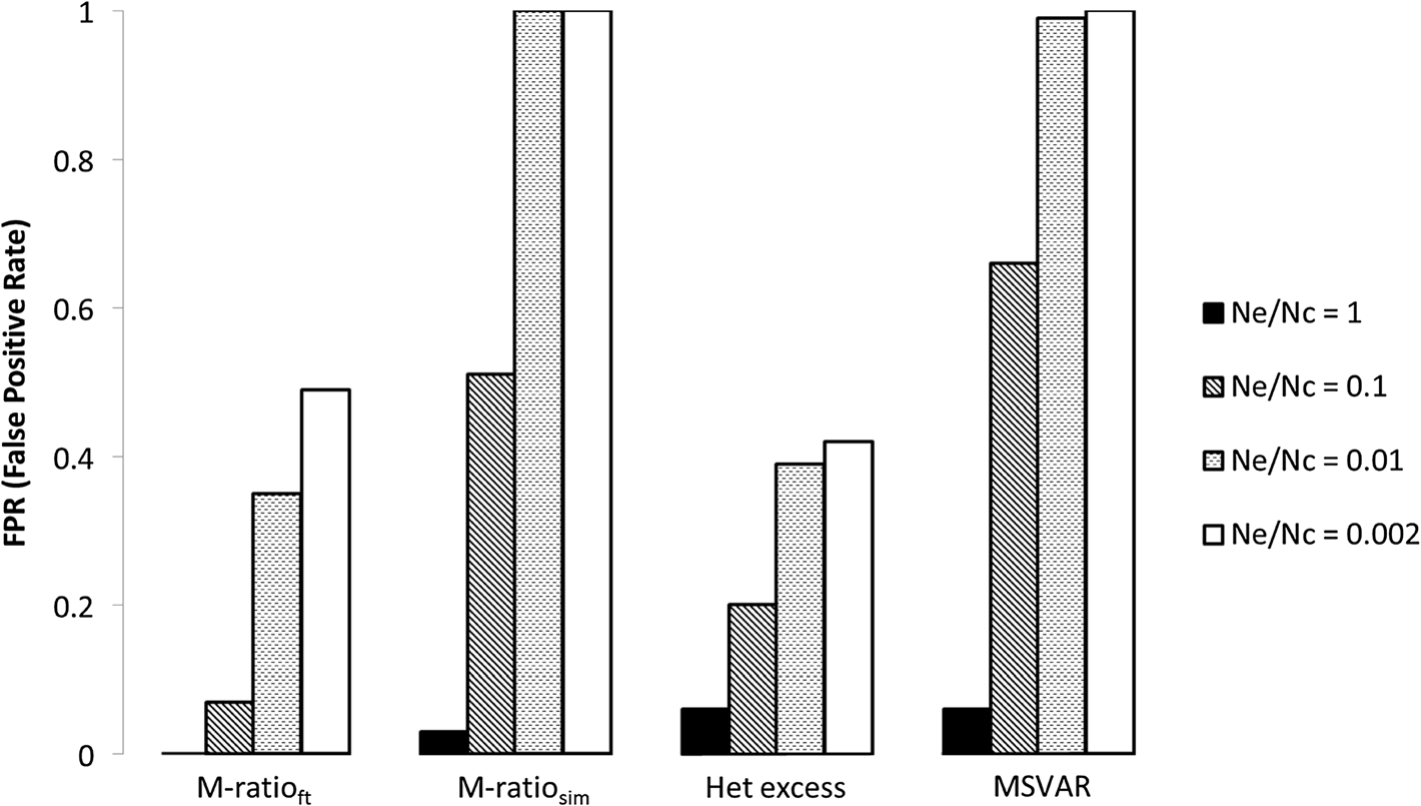
**The false positive rate (FPR) as a function of the ratio *N***_***e***_***/ N***_***c***_**under different statistical approaches.** FPR refers to simulations with *N*_*e*_ = 2500.

**Figure 3:**
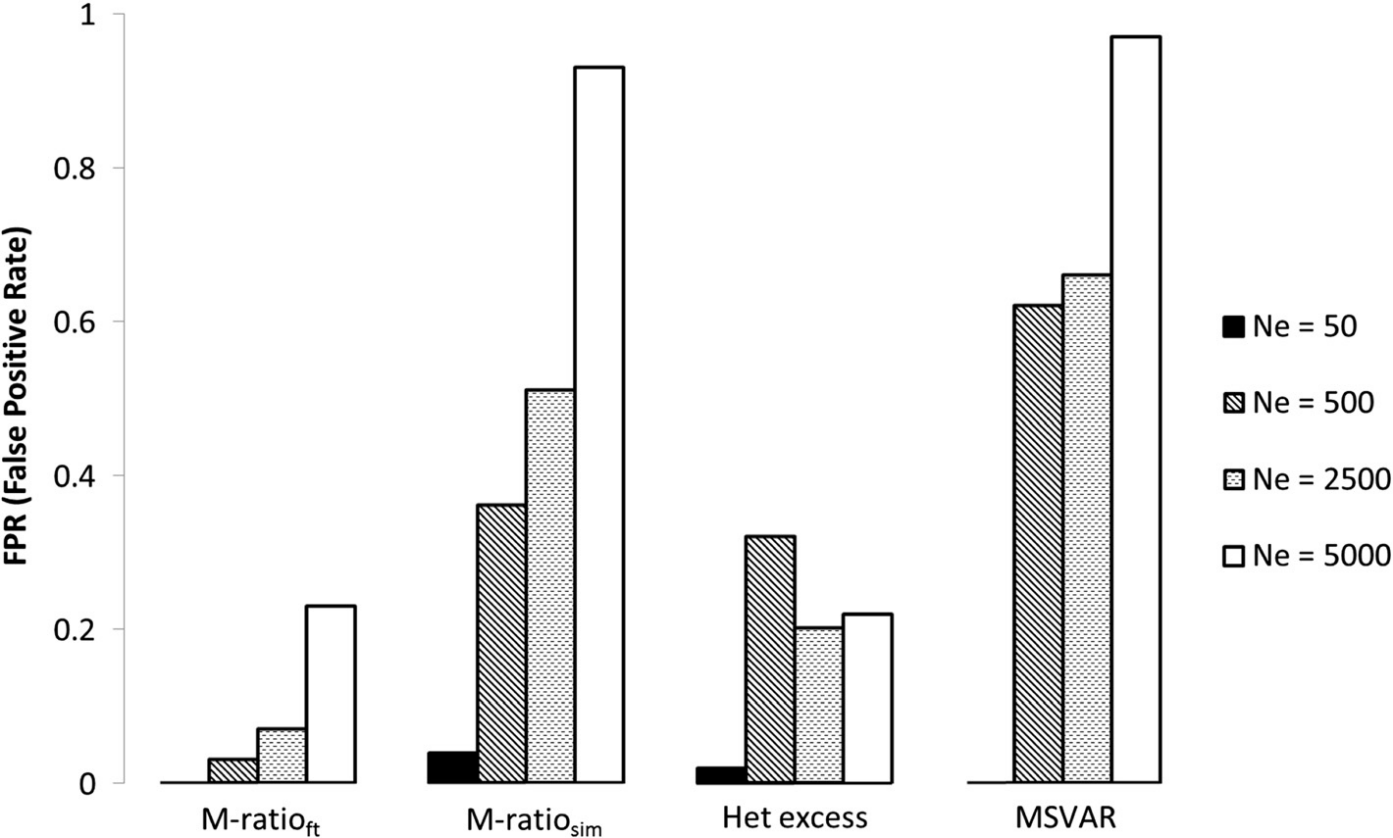
**The false positive rate (FPR) as a function of the effective population size *N***_***e***_**under different statistical approaches.** FPR refers to simulation with *V*_*k*_ =40.

The false positives rate (FPR) clearly increases with *V*_*k*_. With *V*_*k*_=2, FPR for the M-ratio_ft_ test is either 1% or 0% (indicating probably that this criteria is too conservative) and it varies between 2% and 9% using the M-ratio_sim_ test. For the heterozygosity excess test, the FPR with *V*_*k*_=2 is around the nominal 5% or less, and varies between 0% and 14% for the MSVAR analysis (this analysis being more permissive with large values of genetic variation). Very different results are obtained for *V*_*k*_ > 2 (Table 1 and Figure 2), especially for *N*_*e*_ equal or larger than 500 (i.e., when level of polymorphisms is not too low). All or almost all replicates analysed with the M-ratio_sim_ test or with the MSVAR analysis support a bottleneck when *V*_*k*_ ≥ 400 and N_e_ ≥ 500. When the more conservative M-ratio_ft_ test or the heterozygosity excess test are applied, the FPR decreases, but never below 21%. For *V*_*k*_=40, i.e., when the ratio between effective and census size is equal to 0.1, FPR can reach values as high as 93% or 97% in the M-ratio_sim_ test and the MSVAR analysis, respectively. Furthermore, we observe a general trend of FPR to increase with *N*_*e*_ (Table 1 and Figure 3). This pattern, likely related to the overall level of genetic variation available for the tests and to the ratio between the sample size and *N*_*e*_ (which is decreasing when *N*_*e*_ increases), deserves further investigation. In summary, with high variation in reproductive variance, the M-ratio and heterozygosity excess tests produce many false positives, and the probability to detect a spurious bottleneck signal tends to increase with increasing effective population size. MSVAR results are in general similar to those obtained with the M-ratio_sim_ test.

### Additional simulations

*Constrained allelic size – Simulations with Ne=500 and V*_*k*_*=2 or 400.* When microsatellite alleles exhibit strong size restrictions (only 5 alleles with adjacent number of repeats are possible), the fraction of false positives for the heterozygote excess test increased from 1% to 47% when *V*_*k*_ was increased from 2 to 400. This increase in FPR is similar to that observed in the simulations with size-unconstrained loci. However, none of the replicates with high *V*_*k*_ with constrained loci produced small and significant M-ratios. The likely explanation is that a reduced allelic range prevents the opening of gaps in the allelic size distribution. In other words, the M-ratio test does not tend to suggest a false signal of a bottleneck when analyzing size-constrained EST microsatellites.

*DNA sequence polymorphism - Simulations with N*_*e*_*= 500 and V*_*k*_*= 400.* The Tajima’s *D* distribution, centered around 0 for *V*_*k*_ = 2 in case of constant population size and absence of natural selection, is shifted towards positive values, with a mean of 1.24. The FPR, i.e. the fraction of values significantly larger than 0, is 37%. Thus, the Tajima’s *D* statistics is similarly affected by an increased variance in reproductive success, and would frequently support a population decline or balancing selection when *V*_*k*_ >> 2.

## Discussion

In many organisms with high fecundity, the contribution of each individual or pair to the next generation can be highly skewed, with few “winners” (i.e. those who produce many offspring) and many “losers” who do not contribute to the gene pool of the next generation. Under this scenario of Sweepstakes Reproductive Success (SRS) [38], the variance in reproductive success (*V*_*k*_) is larger than assumed by the Wright-Fisher model. Population genetics theory predicts that the ratio of *N*_*e*_ (the effective population size) over *N*_*c*_ (the census population size) rapidly decreases from one as *V*_*k*_ increases. The SRS model is thus considered a likely explanation for the empirical observation that many marine organisms have much lower genetic variation (and therefore *N*_*e*_) than predicted by their very large *N*_*c*_[39].

While the negative relationship between genetic variation and *V*_*k*_ is well known, the effect of *V*_*k*_ on the gene genealogy shape reconstructed from a sample of DNA fragments is yet unclear. It is possible that large *V*_*k*_ values may introduce distortions in this genealogy, in turn distorting the relationships between genetic variation measures. This is relevant as many statistical analyses for identifying deviations from neutrality and demographic stability assume *V*_*k*_=2 and are based on the relationships between genetic variation measures.

We addressed this question by comparing simulated datasets of single populations with different *V*_*k*_ values. Specifically we estimated the impact of large *V*_*k*_ on the results from four statistical tests commonly used to detect population size variation: the M-ratio test, the heterozygote excess test, a test derived from a Bayesian estimate of ancient and current population sizes, and the Tajima’s *D* test. Conceptually, when these tests are applied to neutral markers, the null hypothesis includes demographic stability, no migration and *V*_*k*_=2. Rejection of this hypothesis may be interpreted as population decline, but may be also due to large *V*_*k*_ in isolated, demographically stable populations. This is relevant in conservation genetics as violation of the assumption of low *V*_*k*_ made by these tests can produce incorrect inference, and may suggest incorrect management interventions.

Our simulations show that high *V*_*k*_ can strongly increase the rate of false positives (FPR = type I error = incorrect inference of population decline) for all the tests. Further, the larger *V*_*k*_, the larger the rate. FPR is also dependent, to some extent, on *N*_*e*_ (and thus the level of genetic variation), but this relationship appears test-specific. Based on our results, it appears that the MSVAR method is most prone to errors, followed by the M-ratio with the critical threshold computed by simulations (M-ratio_sim_). The heterozygote excess and M-ratio with the traditional threshold are less prone to false positives when *V*_*k*_ is large and may be preferred for use, if the goal is to reduce type I errors when evidence of large *V*_*k*_ is available. The results we obtained show also that high *V*_*k*_ could cause wrong conclusions when the aim of the analysis is to identify signatures of selection. In particular, the negative *F*_*is*_ values and positive Tajima’s *D* produced in our simulations of neutral markers with large *V*_*k*_ could be misinterpreted as signals of balancing selection.

When *V*_*k*_ is large, a large fraction of siblings is observed every generation. In coalescent terms, several lineages merge in one generation going back in time, producing many short external branches in the gene genealogy and therefore a deviation from the standard Kingman coalescent [27,28]. Allele sharing among individuals will be high and alleles present in one (singletons) or few (rare alleles) individuals will be very low. Considering that bottleneck tests assume the standard Kingman coalescent, or the Wright-Fisher model it approximates, we propose that the excess of short external branches and corresponding deficit of rare alleles could explain the large FPR. In fact, this situation is expected to result in (a) higher heterozygosity than expected based on number of alleles (and thus positive heterozygote excess test and an overall signal of population decline detected by MSVAR), (b) gaps in the microsatellite allele size distribution (and significant M-ratio test) and (c) loss of segregating sites but not substantial reduction in the average pairwise difference (and positive Tajima’s D). We also note that the fraction of siblings and the rate of multiple coalescent events rapidly decreases going back in time (since few lineages survive, additional simulation results not shown); thus, one generation of large *V*_*k*_ can generate large FPR. We also note that the constant population size scenario we simulated appears similar, in its effects, to a scenario of a recent and extreme bottleneck in an additional way, with a small recent effective size producing negative *Fis* values compatible with a population of few individuals [40].

Due to different parameterization of the model of the biological system, our results are not directly comparable with the genetic prediction of recent theoretical models of populations with skewed offspring number and overlapping generations [27,41-45]. These models, which allow for simultaneous multiple coalescent events e.g. [42], suggest that in a “many losers, few winners” situation (high *V*_*k*_), the chances to obtain star-like genealogies and excess of rare alleles, i.e., signatures of population expansion, is increased compared to the *V*_*k*_ =2 case; this is opposite the result obtained in our study. A possible explanation for the discrepancy is the fact that our simulations considered non-overlapping generations, and overlap in generations may provide a buffer against the effects of drift and consequent high allele sharing caused by high *V*_*k*_. Additional efforts should be dedicated to make the results produced by theoretical models with multiple merger and those obtained in our study comparable.

### Practical applications

Certainly, our results suggest that the genetic signature of a bottlenecks should be interpreted with caution when found in species known to have moderate to large variance in offspring number (as for example in the killer whale, [46]), or where large variance in offspring number is suspected (as for example in many marine species, [26]). In the killer whale example, large variance in offspring number was estimated based on parentage analysis and a demographic bottleneck was inferred from genetic data using the statistical approaches we examined in our study; the authors report that it is unclear whether a bottleneck actually occurred. This work, and our simulations, emphasize that robust, widely applicable, powerful alternative methods of detect a bottleneck are still needed.

An alternative to using the standard bottleneck tests for species with large *V*_*k*_ is using computer simulations [16,32]. Summary statistics from observed data can be compared to a distribution of expected values from simulated data created with forward simulations, in simuPOP, spip [47], Nemo [48], cdpop [49] or other software [32]. The distribution of reproductive success and other aspects of the species’ reproductive system can be taken into account in the simulations, allowing the investigator to observe *V*_*k*_ effects on the population genetic signal and, more specifically, generating species-specific null distributions of the bottleneck tests (as the M-ratio statistic) more appropriate for *V*_*k*_ larger than 2. Simulating stable populations, and populations with different intensities of demographic decline, can allow statistical comparison to the observed data (with or without formal approaches like Approximate Bayesian Computation, [50]).

The high FPR we uncover may not present a problem for studies that detect a bottleneck by comparing temporal samples, as comparing a modern sample to museum or ancient samples [23], or comparing to a non-bottlenecked but otherwise similar population [17]. Type I error due to *V*_*k*_ should not be expected to arise because large *V*_*k*_ should affect diversity in both samples. However, this assumes that *V*_*k*_ is constant through time. If census size decreases, *V*_*k*_ may change through time [12], with unknown effects on our ability to detect a bottleneck by comparing ancient and modern genetic variation levels. The increasing use of ancient DNA and prevalence of studies that infer bottlenecks from temporal samples [51], suggests that it will be important to evaluate the effects of high *V*_*k*_ on temporal comparisons.

Finally, considering that our simulations assumed non-overlapping generations, and also considering that effect of drift decreases proportionally to the number of generations that overlap [25,52], we emphasize that our findings should be considered applicable particularly to organisms with non-overlapping generations or short generation times (e.g., annual plants, insects, some fish).

## Conclusions

We have shown that high reproductive variance increases the rate of false positives in four widely used bottleneck detection tests. Failing to detect a genuine bottleneck is widely acknowledged as harmful in conservation. However, given the limited resources and myriad of necessary conservation actions that are required to protect vulnerable species and populations [53], accurate tests are required to identify population bottlenecks with low false positive rates so that resources can be applied where they are needed most. The current study highlights the high type I error rate of bottleneck tests and emphasizes the need for more sophisticated analysis to evaluate conservation status of species with high reproductive variance.

## Competing interests

The authors declare no competing interests.

## Authors’ contributions

MM, CVO, and GB conceived and designed the study, MM and AB performed simulations and data analysis, SH drafted the manuscript and GB and OEG worked on it. All authors examined data, discussed results, contributed to manuscript revision and approved the final draft.

## Authors’ information

All authors are interested in the demographic and genetic dynamics of small or isolated populations, and in the development and testing of statistical approaches to infer population processes from genetic variation data.
